# Baptisia—Typhoid; Aloes—Constipation

**Published:** 1881-04

**Authors:** E. B. Nash

**Affiliations:** Cortland, N. Y.


### BAPTISIA—TYPHOID; ALOES—CONSTIPATION.

BY E. B. NASH, M.D., CORTLAND, N. Y.

KITTIE H——, aged 11 years, dark hair, blue eyes, generally healthy, except a weakness in the lower part of her spine, remaining after an attack of diphtheria which she had two years ago, had, for several days, on returning from school, complained of feeling very weak and tired, and she was forced to lie down and rest—which was very unusual. One evening pain in the head and back followed, and increased until she could not sleep; she was very restless and somewhat delirious all night. When called to visit her the following morning I found the following symptoms: pain in head and back; great drowsiness—can hardly keep her awake long enough to get answers to my questions; *falls asleep while being talked to; face dark red and bloated; eyes congested; tongue trembles when protruded; with a brown streak in the centre; stools very fetid, and passing involuntarily;* urine yellow and offensive; pulse 140. Gave *Baptisia* C.M. (Swan). In the evening there was no change. It was almost impossible to attract her attention; constantly muttering. Gave *Baptisia* 3rd. centesimal. At midnight the change was wonderful; the sensorium was clear, and she recognized me with a smile. Three doses of *Baptisia* had been given. Her mother stated that after the first dose it seemed as though she was being "lifted right out of the stupor." The same remedy was now given in the 200th. and, although the stools continued involuntary for the next twelve hours, the improvement was uninterrupted; she was able to sit up in three days from the first dose of medicine. By comparing the symptoms of this case with those of *Baptisia* in "Guiding Symptoms," it will be seen that this remedy was the simillimum. Why did not the C.M. do as well as the 3rd.? I do not know. The question of dose is an open one. As a rule, I have found this remedy more efficacious in the 30th. potency, in typhoids (when indicated), than in the lower. What is the minimum dose in one case is not in another.

Master P——, aged three years, light hair, complexion and eyes, had been troubled with constipation since birth. At times he was worse than at others, and it was often almost impossible to get an evacuation even with repeated injections of water. The fæces were in lumps, very large and light-colored; there was so much pain attending efforts at stool that he screamed and was covered with sweat, and the mother was often obliged to pick away the hard lumps. He seemed afraid, and avoided letting his parents know, of a desire for stool, as long as possible. After treating him during several of these attacks with *Bry.*, *Sulph.*, *Nux*, *Verat.*, *Cal. carb.*, and *Sepia*, with indifferent success. I found that he oftentimes passed large, hard lumps of fæcal matter involuntarily and apparently unconsciously. *Aloes*, 200th. cured, and there has been no return of the trouble for two years.

According to Hale's theory of dose it would have been necessary to give this remedy *low*, it being secondarily homœopathic to constipation. Such was not the case; nor is it necessary to give *Puls.* low in scanty or delayed menses (when indicated), because, according to the same authority, it is *secondarily* homœopathic to those conditions. (See *U. S. Med. and Surg. Journal*, vol. iii, p. 81). This thing has misled many young practitioners, and made eclectics by the score. It is so much easier to relieve (not cure) a case of constipation by physic (secondarily homœopathic), than it is to always apply the homœopathically indicated remedy.

